# Vitamin D elicits tissue-specific isoform expression in Atlantic salmon muscles

**DOI:** 10.1186/s12864-025-12209-1

**Published:** 2025-11-12

**Authors:** Courtney E. Gorman, Philip McGinnity, C. Darrin Hulsey

**Affiliations:** 1 School of Biology and Environmental Science, University College Dublin, Belfield, Dublin, Ireland; 2 School of Biological, Earth and Environmental Sciences, University College Cork, Cork, Ireland; 3 Marine Institute, Newport, Co. Mayo, Ireland

**Keywords:** Aquaculture, Biofortification, Muscle health, Nutrigenomics, Salmonid, Striated muscle

## Abstract

**Background:**

Accounting for isoforms is likely key to understanding muscle transcriptomic divergence. Muscles offer classic examples of tissues where changes in isoforms alter function and structure in response to stimuli like increased exercise or novel nutrient regimes. To determine how an essential micronutrient alters muscle isoform production, we examined how vitamin D supplementation influences transcription at multiple hierarchical levels across four Atlantic salmon (*Salmo salar*) muscle tissues. Specifically, we investigated whether analyses of differential transcript expression (DTE), differential transcript usage (DTU), and alternative splicing (DAS) recovered different responses to vitamin D compared to differential gene expression (DGE) alone.

**Results:**

Vitamin D modulates salmon muscle transcription at the level of the gene, transcript, and splice junctions in all four muscle tissues. However, the strongest effects at all levels were found in the heart. There was little overlap among significant genes found at the gene, transcript, and splicing levels and the distribution of isoforms per significant gene varied across DGE, DTE, and DTU, indicating that each method tends to identify unique sets of genes. For example, we found that several myosin light chain kinase isoforms were particularly impacted by vitamin D in the heart, but many of the genes exhibited isoforms that were differentially expressed in opposing directions and were thus masked in DGE analysis. We also identified myofibrillar genes that are impacted by vitamin D at all levels of gene regulation, demonstrating that vitamin D impacts many structural proteins that are directly involved in striated muscle contractions.

**Conclusions:**

Vitamin D influences several muscle tissue types across multiple levels of transcriptomic divergence. Further, the limited overlap among significant genes found at the gene, transcript, and splicing levels suggests examining only DGE can mask important genes that show differential effects at other levels. Myofibrillar gene isoforms that directly influence muscle contractions in critical organs like the heart could provide an especially fruitful avenue of additional investigation into the transcriptional impacts of vitamin D. Together, these findings clarify how vitamin D influences muscle differentiation, health, and function.

**Supplementary Information:**

The online version contains supplementary material available at 10.1186/s12864-025-12209-1.

## Background

Genes are often transcribed into multiple isoforms through alternative splicing, whereby exons and other amino acid coding regions from the same gene are included or excluded to produce different mRNA transcripts. This mechanism enables single genes to generate a diverse array of proteins [1, 2]. For instance, more than 95% of multi-exonic human pre-mRNAs exhibit variation in splicing to produce multiple mRNAs [3, 4]. These isoforms can have overlapping, tissue-specific, or even contradictory functions [5]. Much of our current understanding of alternative splicing comes from pathological contexts [6], such as cancer or heart failure [7], where splicing dysregulation plays a clear role. This disease-centered focus could bias our understanding of the functionality and relative importance of alternative isoforms. In contrast, much less is known about the generation of alternative isoforms in the context of improved physiological health, such as when nutritional status is improved. For example, the role of essential nutrients like vitamin D in regulating alternative splicing remains poorly characterized, despite its known effects on musculoskeletal systems and overall health [8, 9]. To begin addressing this gap, we investigated how vitamin D supplementation affects gene and transcript-level expression across muscle tissues in Atlantic salmon (*Salmo salar*), a vertebrate model that requires vitamin D for normal physiological function and responds positively to supplementation [10].

Muscle tissues offer a powerful context for studying splicing responses to nutrient availability because many structural proteins, particularly myofibrillar proteins that form the cross-bridges essential to striated muscle contraction, exist in multiple isoforms that contribute to muscle diversity and function [11–13]. The contractile and energetic properties of muscle fibers are determined not only by the relative abundance of myofibrillar proteins, but also by the presence of specific myofibrillar protein isoforms, such as those of genes like myosin that play a central structural role during muscle contraction [14, 15]. Slight variations among myofibrillar protein isoforms can generate a diversity of structural and functional properties through alternative splicing of the same gene, and most myofibrillar proteins exist in a number of isoforms [12]. These isoforms are often tissue-specific [13, 15, 16] and can be influenced by environmental factors, including dietary vitamin D [17].

Vitamin D is known to play a key role in general musculoskeletal health [8, 9] and accumulating evidence from vertebrate models suggests it also regulates skeletal muscle development and regeneration [18, 19], including through direct effects on the expression of contractile proteins [17, 20]. In addition to its transcriptional effects, vitamin D has been shown to influence alternative splicing, particularly in metabolism-related genes like *CYP24A1*, which encodes the vitamin D-catabolic enzyme 24-hydroxylase [21]. These findings suggest that vitamin D could modulate isoform production in addition to its role in influencing overall gene expression. However, despite its well-established role in muscle physiology, whether vitamin D affects the splicing of contractile protein genes specifically, is less clear. Even less is known about how this essential nutrient may shape isoform diversity across multiple muscle tissues simultaneously, despite broad evidence that vitamin D influences vertebrate health systemically.

Alternative gene expression and isoform production in response to the alteration of physiological conditions is most frequently examined in single tissues. For instance, investigations into the effects of vitamin D on muscle have often been conducted in humans [22–24]. A focus on human subjects presents extensive experimental challenges to the parallel examination of multiple muscles due to the need for invasive and destructive sampling. However, new animal models like Atlantic salmon (*Salmo salar*) have recently emerged as useful for investigating the intricate dynamics of gene expression in vertebrates in response to changes in nutrients like vitamin D [10]. Advantageously, variation in salmonid fish gene expression is increasingly being investigated in both natural and aquaculture settings [25, 26]. Salmon is also one of the most economically and nutritionally important fish species used in aquaculture and, like humans, exhibits substantial natural variation in vitamin D levels [27]. Unlike humans, salmon acquire vitamin D exclusively through their diet [28, 29], allowing for extensive experimental manipulation of vitamin D levels in their muscles via feed supplementation [10, 27]. This enables the examination of the effects of vitamin D on isoform diversity, particularly the variability of RNA isoforms across multiple muscle tissues, in this commonly farmed fish. In addition, manipulation of the composition of muscle myofibrillar proteins in salmon could influence commercially available meat quality, texture, and flavor [30]. Thus, understanding how vitamin D influences muscle myofibrillar proteins has implications for fish aquaculture as well as our general understanding of isoform divergence across muscles.

Muscles differ substantially in how they function and could respond in highly divergent ways to changes in the concentration of important nutrients like vitamin D. Different muscle types perform distinct roles in movement, growth, and overall health [31], and vitamin D could have many tissue-specific effects on isoform expression. Salmon, like humans, possess three major muscle types: (1) skeletal muscle produces movement and is the musculature eaten as fish filets; (2) smooth muscle found in organs like the stomach; and (3) cardiac muscle which drives the heart’s contractions to maintain circulation. Further, skeletal muscles in the head that contribute to the trophic apparatus are developmentally differentiated from the more commonly studied trunk muscles [32–34] and could allow the evaluation of whether various types of skeletal muscle respond differently to vitamin D supplementation. All skeletal muscles appear striated as they are made of individual fibers, composed of primarily actin and myosin, and organized into a spindle [31]. Cardiac muscle is also striated, although it is comprised of individual cardiomyocytes and contains both cytoskeletal and contractile elements that are connected through intercalated discs [35]. Myofibrillar proteins form the molecular components of the cross-bridges that give rise to the visible striations of muscles cells in both skeletal and cardiac muscles. Smooth muscles also contain actin and myosin fibers, but these fibers are arranged in sheets and are not striated like skeletal muscles [31]. While vitamin D has been documented to regulate the expression of contractile protein genes in skeletal muscle [17, 20], much less is known about how vitamin D may impact a diversity of myofibrillar proteins in cardiac or smooth muscle. For instance, if vitamin D were to heavily alter myofibrillar isoforms in cardiac muscle, it would provide evidence that vitamin D directly influences the contractile machinery of the beating heart. Comparisons of how differential expression varies among muscle types should allow us to determine not only how vitamin D differentially influences individual types of muscles, but also at what level vitamin D induced transcriptomic changes in gene expression are altered.

**Fig. 1:**
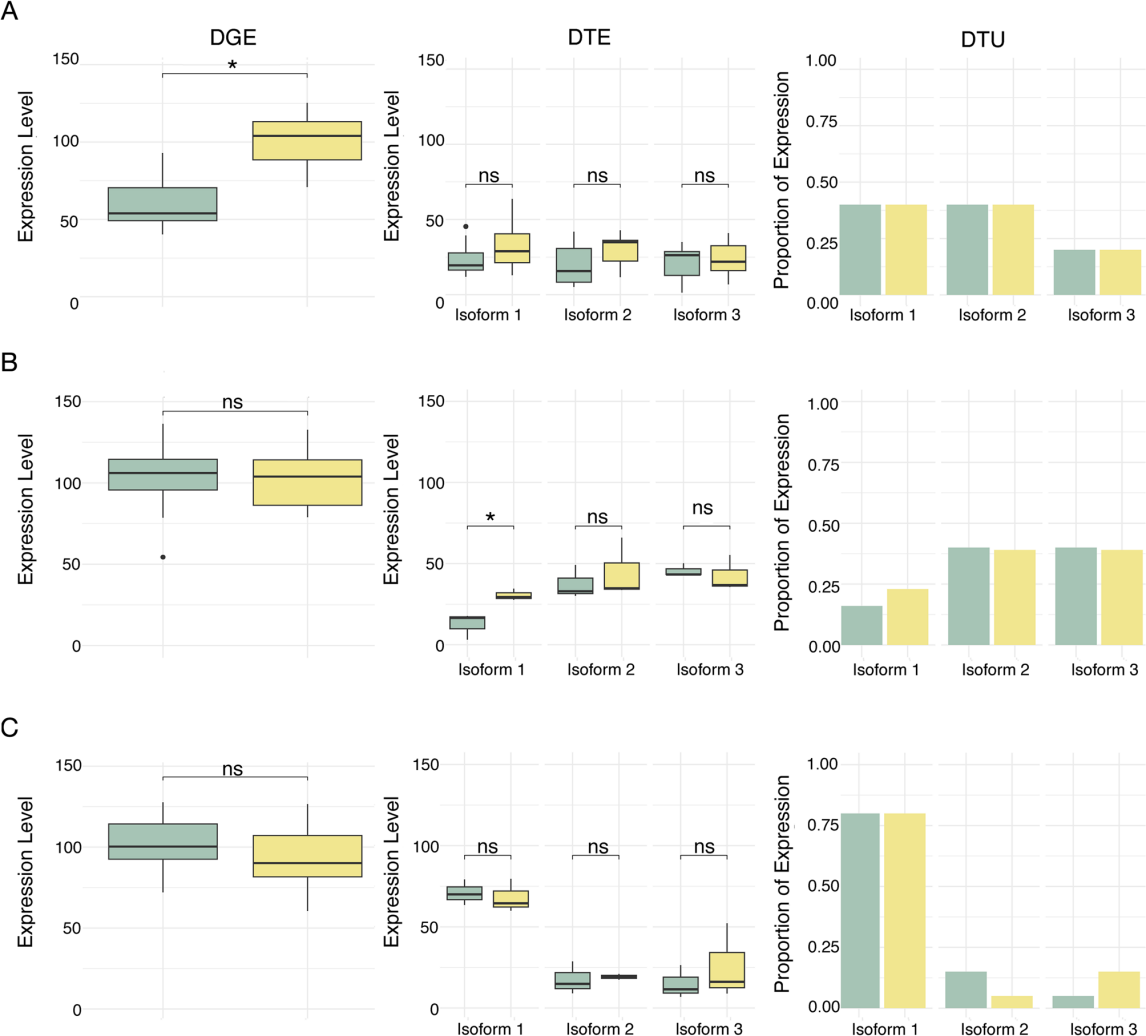
Conceptual diagram illustrating scenarios where significant transcriptional differences are detected in only one type of analysis. Green boxes and bars indicate the control and yellow indicate the treatment. **A **Significant differential gene expression (DGE) but non-significant differential transcript expression (DTE) and differential transcript usage (DTU). When analyzed collectively in a DGE analysis, the overall upregulation of a gene in the treatment samples (e.g., higher vitamin D) is significantly higher. However, although the expression of each of the three expressed isoforms is slightly higher in the treatments than they are in the control, their difference in expression does not reach the significance threshold for any of the three individual isoforms. Since the relative proportions of the three isoforms remain unchanged, DTU is also non-significant. **B **Significant DTE but not DGE and DTU. While overall gene expression is slightly higher in the treatment than in the control, it does not reach significance for DGE. However, isoform 1 is significantly upregulated in the treatment, leading to a significant result for DTE. DTU remains non-significant because there is no significant shift in the relative proportions of isoforms. **C **Significant DTU but non-significant DGE and DTE. Gene-level expression differences are not significant. While isoform expression levels vary, the small changes in expression do not reach the significance threshold for DTE. However, DTU is significant because the relative proportions of isoform 2, that decreases, and isoform 3, that increases, have together shifted significantly

The transcriptomic impacts of vitamin D on salmon muscle associated genes can be characterized across at least three hierarchical levels of differentiation (see Table 1 for an example workflow that outlines the approaches applied in the current study). Differential gene expression (DGE) examines changes in the cumulative expression of all transcripts produced by a single gene (Fig. 1). This whole gene level DGE is the most commonly investigated insight into the overall changes in gene activation or suppression within muscle tissue in response to vitamin D [10, 36, 37]. Some genes also only generate a single isoform. However, many genes often produce multiple transcripts or isoforms, and we might expect the multiple isoforms produced from these genes to each have the potential to exhibit distinct responses to vitamin D. DGE alone likely does not fully capture these isoform-level changes [38]. To address this, differential transcript expression (DTE) can be used to identify changes in the expression of individual isoforms. For instance, it is possible that the amount that a single isoform from a particular gene is expressed could vary in response to vitamin D, but when grouped with all the other isoforms from that gene, the change in overall gene expression would not meet the threshold for DGE (Fig. 1). Also, at the level of change in isoform expression, differential transcript usage (DTU) can detect shifts in the relative proportions of different isoforms transcribed from a single gene [39, 40]. For a single gene, if one isoform is upregulated in response to vitamin D and another isoform decreases in that exact proportion, that whole gene might have zero detectable changes in DGE but substantially different DTU (Fig. 1). Finally, differential alternative splicing (DAS) analysis can be used to detect variability in specific splicing events in response to vitamin D across tissues. DAS detected splicing events could underlie most isoform switches, especially if for example different isoforms differ by only a single individual exons [41, 42]. These different hierarchical levels could readily nest neatly within one another. Whole gene level expression measures such as DGE could effectively capture most of the underlying transcriptional variation in isoforms as well as the particular splicing events that generate these isoforms. Alternatively, muscle tissue types that show extensive DGE as previously observed for the heart in response to vitamin D [10] might vary little in DTE or DTU. Further, even within the same tissue, the complexity of isoform variation could provide a completely divergent view of changes in how vitamin D modulates particular expression pathways. Our understanding of how gene expression is altered in muscles in response to nutrients like vitamin D could substantially change when we account for differences due to alternative splicing.

**Table 1:** Overview of transcriptomic analyses applied to RNA-seq data in the current study

Analysis	Level of Resolution	Biological Insight	Bioinformatic Tools
Differential Gene Expression (DGE)	Gene	Gene-level expression changes	Salmon [43], tximport [44], DESeq2 [45]
Differential Transcript Expression (DTE)	Transcript	Transcript-level expression changes	Salmon [43], tximport [44], DESeq2 [45]
Differential Transcript Usage (DTU)	Transcript	Shifts in transcript proportions	Salmon [43], tximport [44], DRIMSeq [39], stageR [46]
Differential Alternative Splicing (DAS)	Splicing event	Changes in specific splicing events between conditions	rMATS [41]

**Fig. 2:**
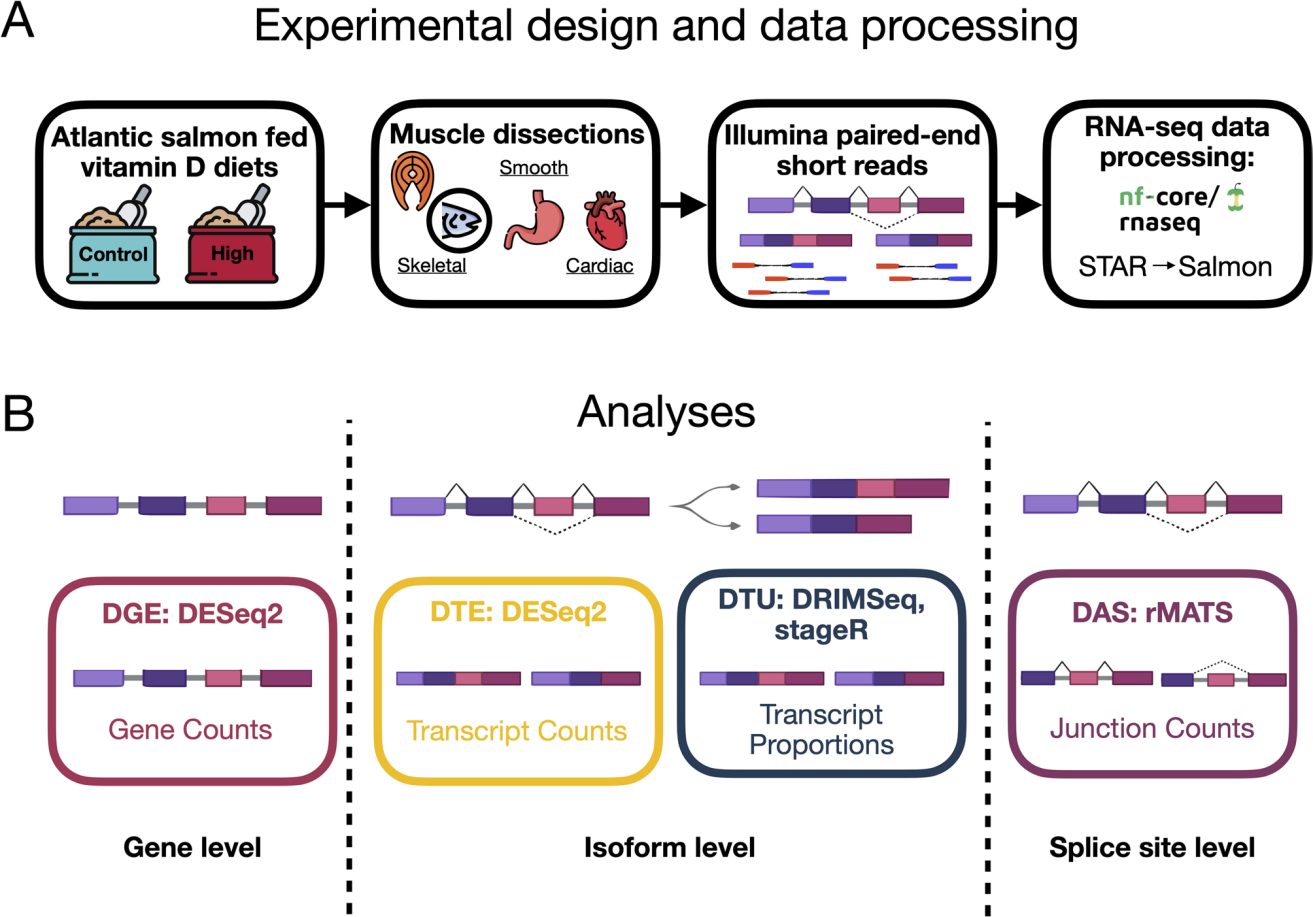
Overview of experimental workflow. Salmon were raised in experimental ponds and the level of vitamin D in their diets (Control and High) was manipulated for six months. Four major muscle tissues, (1) axial skeletal (filet), (2) craniofacial (adductor mandibulae), (3) smooth (stomach), and (4) cardiac (heart), were sampled to determine how vitamin D impacts transcription in salmon muscles. Analyses of multiple levels (gene, isoforms, and splice sites) of transcriptomic divergence (DGE, DTE, DTU, and DAS) were performed for all four muscles in fish from the vitamin D manipulations

By experimentally manipulating the extent of vitamin D supplementation in juvenile *Salmo salar*, we determined how vitamin D alters multiple aspects of transcription in four muscles tissues. We asked whether vitamin D supplementation influenced transcript expression, usage, or alternative splicing events in any of the four muscles. Additionally, we highlighted affected proteins known to be involved in striated muscle contraction (i.e., myofibrillar and myofibrillar-associated proteins). Then, we compared these results to an examination of differential gene expression to assess which impacts of vitamin D on muscles may be overlooked when grouping isoforms at the gene level. Specifically, we performed transcriptomic analyses including the examination of DTE, DTU, and DAS across four muscle tissues in response to vitamin D supplementation and compared the results to an analysis of DGE alone (see Fig. 2 for overview). This analysis of the transcriptomic effects of vitamin D induced isoforms in salmon at multiple hierarchical levels should provide more robust inferences of this important vitamin’s simultaneous impacts on multiple types of vertebrate muscle.

## Methods

### Experimental design and sampling

Atlantic salmon (*Salmo salar*) from both a Norwegian aquaculture strain and a captive bred Irish strain, obtained from stocks at the Marine Institute Newport Research Facility in County Mayo, Ireland, were used to produce juveniles for the experiment. Multiple male and female parents from each strain generated the alevins, which were raised to the free-swimming fry stage and then placed in outdoor experimental enclosures at the Marine Institute Newport Research Facility. These circular enclosures (3.6 m diameter) were supplied with natural freshwater drawn from a lake above the experimental enclosures, which was effectively replaced every few minutes. Depths were maintained at 30 to 45 cm and ambient temperatures ranged between 5 and 20 ◦C during the six month experimental trial.

Once feeding commenced, fry were maintained on a commercial diet for one week. After this acclimation period, fry were assigned to one of two experimental diets, both based on commercial feed containing a vitamin-mineral premix that included a baseline level of vitamin D_3_ (cholecalciferol). To create the treatment diets, either 0 ng/g or 1000 ng/g vitamin D_3_ was added to the base feed. This resulted in final measured concentrations of 729 ng/g in the control diet and 1779 ng/g in the high vitamin D₃ diet [10]. These concentrations were previously shown to affect muscle vitamin D accumulation and gene expression [10], and were used here to investigate transcript-level regulatory responses via RNA-seq.

To sample the tissues for RNA-sequencing, the fish were sacrificed with an overdose of MS222 and muscles dissected during a single week following six months of experimental diet consumption. We subsequently dissected approximately 1 g of each tissue from the dorsal right side of the fish corresponding to frequently consumed salmon filets (axial skeletal muscle), the adductor mandibulae (craniofacial skeletal muscle), the stomach (smooth muscle), and heart (cardiac muscle) from three individuals of each strain per treatment (2 vitamin D levels × 4 muscle tissues × 3 individuals × 2 salmon strains; *n* = 48). We selected these four muscles because they represent distinct functional classes that differ developmentally, structurally, and physiologically. This allowed us to assess whether vitamin D supplementation elicits both shared and tissue-specific transcriptomic responses across major muscle types. As the individuals were juveniles at the time of sampling and therefore not sexually dimorphic, sex was not determined. Images of a representative dissection are included in Supplementary File 1. Following dissection, the tissues were individually stored in RNAlater (Sigma-Aldrich) in labeled 1.5mL tubes prior to sequencing.

RNA samples were extracted from the four Atlantic salmon muscle tissues and were sequenced on the Illumina NovaSeq X Plus (PE 150) platform (Novogene, Cambridge, UK) to generate approximately 12G raw data per sample. The RNA-seq data are available at the NCBI Sequence Read Archive under accession number PRJNA1160017.

## RNA-seq data processing

Raw RNA-seq data were processed using nf-core/rnaseq v3.14.0 of the nf-core collection of workflows [47], utilizing reproducible software environments from the Bioconda [48] and Biocontainers [49] projects. The pipeline was executed with Nextflow v24.04.4 [50] on Sonic, a high-performance computing cluster located at University College Dublin, Ireland. In brief, raw FastQ files were subsampled to 1 million reads and Salmon Quant was used to infer strandedness. Quality and adapter trimming was then performed on FastQ files using Trim Galore!, a wrapper tool around Cutadapt [51] and FastQC [52]. The pipeline then used STAR [53] to map the raw FastQ reads to the reference genome (Ssal_v3.1), project the alignments onto the transcriptome, and to perform the downstream BAM-level quantification with Salmon [43]. The genome fasta and gtf file were then used to generate the transcripts fasta file, which was further utilized to build the Salmon index.

### Differential gene expression

Although a differential gene expression analysis of these data was performed previously [10], we re-analyzed the data here using Salmon [43] as part of the upstream Nextflow pipeline. This allowed the current DGE results to be directly comparable with all downstream isoform-focused analyses. Differential expression analyses were performed separately for each muscle tissue, ensuring that observed differences within each analysis could be attributed to vitamin D treatment rather than tissue-type effects. We used tximport [54] to import the quantification data into R. Genes were filtered to retain only those with a total count greater than or equal to 10 across at least three samples. Differential expression analysis of two conditions (high versus control), each with six biological replicates, was conducted using the DESeq2 R package (v1.44.0) [45]. Each set of six replicates included three individuals from each of two Atlantic salmon strains. These strains were included to increase genetic diversity in the experiment and were grouped for analysis based on prior evidence showing no significant differences in vitamin D accumulation between strains in filet muscle [10]. The DESeq2 design model included vitamin D treatment as the main effect and controlled for strain as a batch effect. P-values were adjusted for multiple testing using the Benjamini-Hochberg procedure to control the false discovery rate [55]. Genes with an adjusted p-value ≤ 0.05 were designated as differentially expressed. To improve effect size estimation, log_2_ fold changes (log_2_FC) were shrunk using the lfcShrink() function with the apeglm method [56]. No log_2_FC threshold was applied during differential expression analysis in order to retain all genes with statistically significant responses to vitamin D treatment.

### Differential transcript expression

Differential transcript expression analysis was conducted separately for each muscle tissue using DESeq2 (v1.44.0) in R [45]. Transcript-level quantification data from Salmon [43] were imported using the tximport function [54]. A DESeqDataSet object was created using DESeqDataSetFromTximport, and transcripts were filtered to retain only those with a total count greater than or equal to 10 across at least three samples. The DESeq2 design included vitamin D treatment as the primary condition and accounted for strain as a batch effect. Differential expression analysis was performed using the DESeq function with default parameters, comparing high and control conditions. Transcripts with an adjusted p-value ≤ 0.05 were designated as differentially expressed. To improve effect size estimation, log_2_FC changes were shrunk using the lfcShrink() function with the apeglm method [56].

### Differential transcript usage

Differential transcript usage analysis was performed for each muscle tissue. Transcript-level abundance was quantified using Salmon [43] and imported into R with tximport using the scaledTPM method to obtain count-scaled transcript abundances for DTU analysis. To map transcripts to genes, we used a custom-built transcript database created with makeTxDbFromGFF from the txdbmaker package [57], based on the annotation file (Salmo_salar.Ssal_v3.1.112.gtf). This TxDb object linked transcript and gene identifiers, which allowed for the organization of transcript-level counts by gene. We then performed DTU analysis using DRIMSeq [39]. Filtered samples and counts were combined in a dmDSdata object. Counts were filtered to retain transcripts with a minimum of 10 reads in at least six samples. Additional filters required transcripts to have a minimum relative abundance of 0.1 in at least six samples, and genes to have a total count of at least 10 in all samples. A design matrix was created with vitamin D treatment (high or control) as the condition, and the dmPrecision, dmFit, and dmTest functions were applied sequentially to estimate the model parameters and test for differential transcript usage. The results function was used to build a test result table. This table included both a single p-value per gene to test whether there was any differential transcript usage for the gene, and a single p-value per transcript, which tested whether the proportions of transcripts changed for the gene. To improve the overall false discovery rate across transcripts within each gene, we used stageR to perform stage-wise FDR control [46] with an overall false discovery rate target of 5%.

### Differential alternative splicing

To identify and quantify support for different splicing events that generate isoforms in response to vitamin D, we conducted a differential alternative splicing analysis for each muscle tissue. Unlike DGE, DTE, or DTU, alternative splicing analysis allows for the identification of specific splicing events that contribute to transcript diversity [41]. The software rMATS was used to define differential alternative splicing events by computing and comparing the inclusion level of each alternative splicing event between the two RNA-seq datasets (high and control). Differential alternative splicing events with an FDR-adjusted p-value of < 0.05 were considered significant.

### Gene Ontology (GO) enrichment of DGE, DTE, DTU, and DAS

Gene Ontology (GO) enrichment was performed using the gprofiler2 R package (v. 0.2.3) [58] with the organism code *ssalar*. We tested four gene sets: (1) all significant genes from the differential gene expression (DGE) analysis, (2) all significant genes from the differential transcript expression (DTE) analysis, (3) genes significant in DGE but not in DTE (DGE only), and (4) genes significant in DTE but not in DGE (DTE only). GO enrichment analyses were not performed on genes with significant differential transcript usage or alternative splicing events due to an insufficient number of genes. Gene sets were defined by unique Ensembl gene identifiers from each analysis. For each set, enrichment was assessed across all three GO ontologies, Biological Process (BP), Molecular Function (MF), and Cellular Component (CC), using the Benjamini-Hochberg false discovery rate (FDR) correction (correction_method = “fdr”) and a significance threshold of *p* < 0.05 (user_threshold = 0.05). Terms were retained if they had a term size between 5 and 1000 and an intersection size ≥ 3 genes from the tested set.

### Distribution of the number of isoforms in DGE, DTE, and DTU

Genes that produce multiple isoforms could more commonly exhibit DTE or DTU. To determine the distribution of the number of isoforms per gene for genes with significant DGE, DTE, and DTU we first summarized the number of transcripts per gene for all genes in the Ssal_v3.1 GTF file. Notably, no genes in this annotation were listed with more than ten isoforms, suggesting a possible technical cap in the reference file. To avoid potential bias caused by this apparent cap (e.g., the possibility that some genes with more than ten isoforms could be assigned to have only ten isoforms), we excluded genes annotated with exactly ten transcripts. We then determined the number of transcripts per gene for each of the lists of genes with significant DGE, DTE, and DTU and calculated the mean number of transcripts per gene for the significant genes in each analysis.

### Identification of genes linked to myofibrillar proteins

To generate a list of genes in the Atlantic salmon genome that are likely linked to myofibrillar proteins, we used an overview of the human muscle cytoskeleton [13] to make a list of known myofibrillar proteins. We then manually searched the Atlantic salmon Ensembl database for each term on June 24, 2024, and recorded the Ensembl gene ID, gene name, and gene description for each gene that matched a muscle cytoskeleton component. This resulted in a list of 563 genes that are myofibrillar or myofibrillar-associated genes [see Supplementary File 2].

## Results

### Transcripts expressed in each muscle

The number of detected genes and transcripts was similar among the muscle types (Table 2). However, despite cardiac muscle having roughly the same number of genes and transcripts expressed as the other muscle types, it had a much higher number of transcripts that were expressed only in the heart and not the other muscles (~ 10,000 heart-specific transcripts versus ~ 1000 tissue-specific transcripts for the other muscles; Table 2).

**Table 2:** Summary of transcripts expressed in each muscle in control and high vitamin D salmon. Transcripts were filtered to retain only those with a total count greater than or equal to 10 across at least three samples and were counted if they were inferred to be expressed with transcripts per kilobase million values greater than or equal to 1.0. For each muscle under both the control and high vitamin D conditions, the total number of expressed transcripts and genes, as well as the number of transcripts uniquely expressed in that tissue compared to other muscles, are reported

	Control			High		
	Genes	Transcripts	Tissue-specific transcripts	Genes	Transcripts	Tissue-specific transcripts
Filet	25,638	42,653	1261	26,262	43,751	1293
ADMs	24,478	39,027	729	25,554	41,435	1037
Stomach	27,150	45,541	1896	26,375	43,192	1381
Heart	28,330	50,725	10,115	28,379	50,497	9436

### Differential gene expression

Our differential gene expression (DGE) results across all four muscles were consistent with our previous findings, despite different analytical methods [10]. In the filet, two genes were significantly upregulated, both with log_2_ fold changes (log_2_FC) greater than one, indicating at least a two-fold increase in expression in the high vitamin D group compared to the control (Supplementary File 3.1). In the adductor mandibulae we identified five downregulated genes and three upregulated genes (Supplementary File 3.1). Two of the downregulated genes and all three upregulated genes had an absolute log_2_FC greater than one. The stomach showed five downregulated genes and two upregulated genes, with two of the downregulated and both upregulated genes exceeding an absolute log_2_FC of one (Supplementary File 3.1). None of the differentially expressed genes in the filet, adductor mandibulae, or stomach were known myofibrillar or myofibrillar-associated genes.

Consistent with Gorman et al. [10], we found that the heart exhibited the largest transcriptomic response to vitamin D supplementation. With 483 genes significantly downregulated and 396 upregulated, the number of differentially expressed genes in the heart in response to vitamin D supplementation were several orders of magnitude greater than the other muscles (Supplementary File 3.1). Of the differentially expressed genes in the heart, 41 downregulated and 25 upregulated genes had an absolute log_2_FC exceeding one. Twelve differentially expressed genes in the heart were known myofibrillar or myofibrillar-associated genes, including two copies of *acta1* (actin alpha 1), *cby1* (chibby1), *desma* (desmin a), *flncb* (filamin C, gamma b (actin binding protein 280)), *lmo2* (LIM domain only 2), *mylk4b* (myosin light chain kinase family, member 4b), *pdlim7* (PDZ and LIM domain 7), *pfkpa* (phosphofructokinase, platelet a), *sntb1* (syntrophin, basic 1), *unc45a* (unc-45 myosin chaperone A), and *vcl* (vinculin).

### Differential transcript expression

We also performed a differential transcript expression analysis, in part because many genes that produce multiple isoforms are not found to show differential expression at the level of the entire gene. In the filet, we identified 43 differentially expressed transcripts, with 22 transcripts downregulated and 21 upregulated (Supplementary File 3.2). Two downregulated and 16 upregulated transcripts had an absolute log_2_FC greater than one. None of the differentially expressed transcripts in the filet were associated with known myofibrillar genes. In the adductor mandibulae, we found 65 differentially expressed transcripts, with 44 downregulated and 21 upregulated (Supplementary File 3.2). Eight of the downregulated and nine of the upregulated transcripts had an absolute log_2_FC exceeding one. Three of the genes that exhibited DTE in the adductor mandibulae were myofibrillar or myofibrillar-associated genes: *itgav* (integrin, alpha V), *lmnb1* (lamin B1), and *myh10* (myosin heavy chain 10). The stomach had 40 differentially expressed transcripts, with 18 downregulated and 22 upregulated, of which four downregulated and six upregulated had an absolute log_2_FC greater than one (Supplementary File 3.2). None of the differentially expressed transcripts in the stomach were associated with known myofibrillar genes. As with the DGE results, the most pronounced effects of vitamin D were seen in the heart, where 609 transcripts were downregulated and 534 were upregulated (Supplementary File 3.2). Of these, 88 downregulated and 59 upregulated transcripts had an absolute log_2_FC exceeding one. In the heart, there were 23 transcripts from 17 genes that were myofibrillar or myofibrillar-associated, including two salmonid-specific duplicate gene copies of *acta1*, *flncb*, and *mylk3* (myosin light chain kinase 3).

### Many genes with DTE were not significant in DGE analysis

**Fig. 3:**
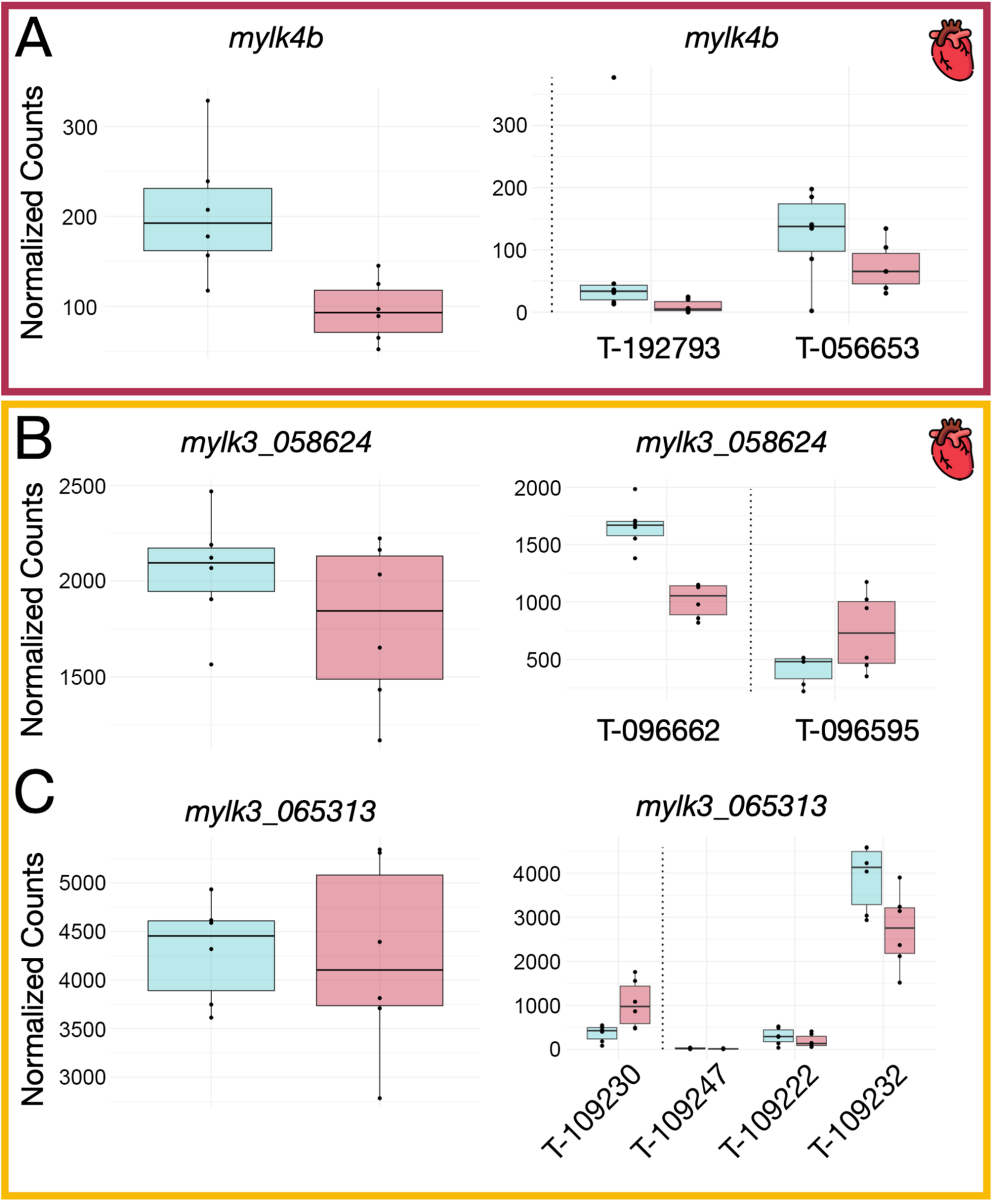
Transcriptional diversity of *mylk* genes in the heart. DGE and DTE for *mylk4b* and two genomic copies of *mylk3* are shown with blue boxes plotted for the Control and red boxes indicating the High vitamin D salmon. The transcript IDs are abbreviated with T- and the last six digits of the Ensembl ID. Transcripts that were significantly differentially expressed appear to the left of the dotted lines (no transcripts were significantly differentially expressed in A). The gene *mylk4b* (**A**) had significant DGE (log_2_FC = −0.85, adjusted p-value ≤ 0.05) but non-significant DTE, while the two copies of *mylk3* (**B**, **C**) had significantly differentially expressed transcripts (B: log_2_FC = −0.67, adjusted p-value ≤ 0.05; C: log_2_FC = 1.22, adjusted p-value ≤ 0.05) but non-significant DGE

There were 645 genes that exhibited DTE but not DGE across the four muscles, including 12 myofibrillar or myofibrillar-associated proteins (three in the adductor mandibulae and nine in the heart; Supplementary File 3.2). For example, two myosin light chain kinase 3 (*mylk3*) genes (i.e., a gene duplicate) had differentially expressed transcripts but no significant DGE in the heart (Fig. 3).

### Differential transcript usage

**Fig. 4:**
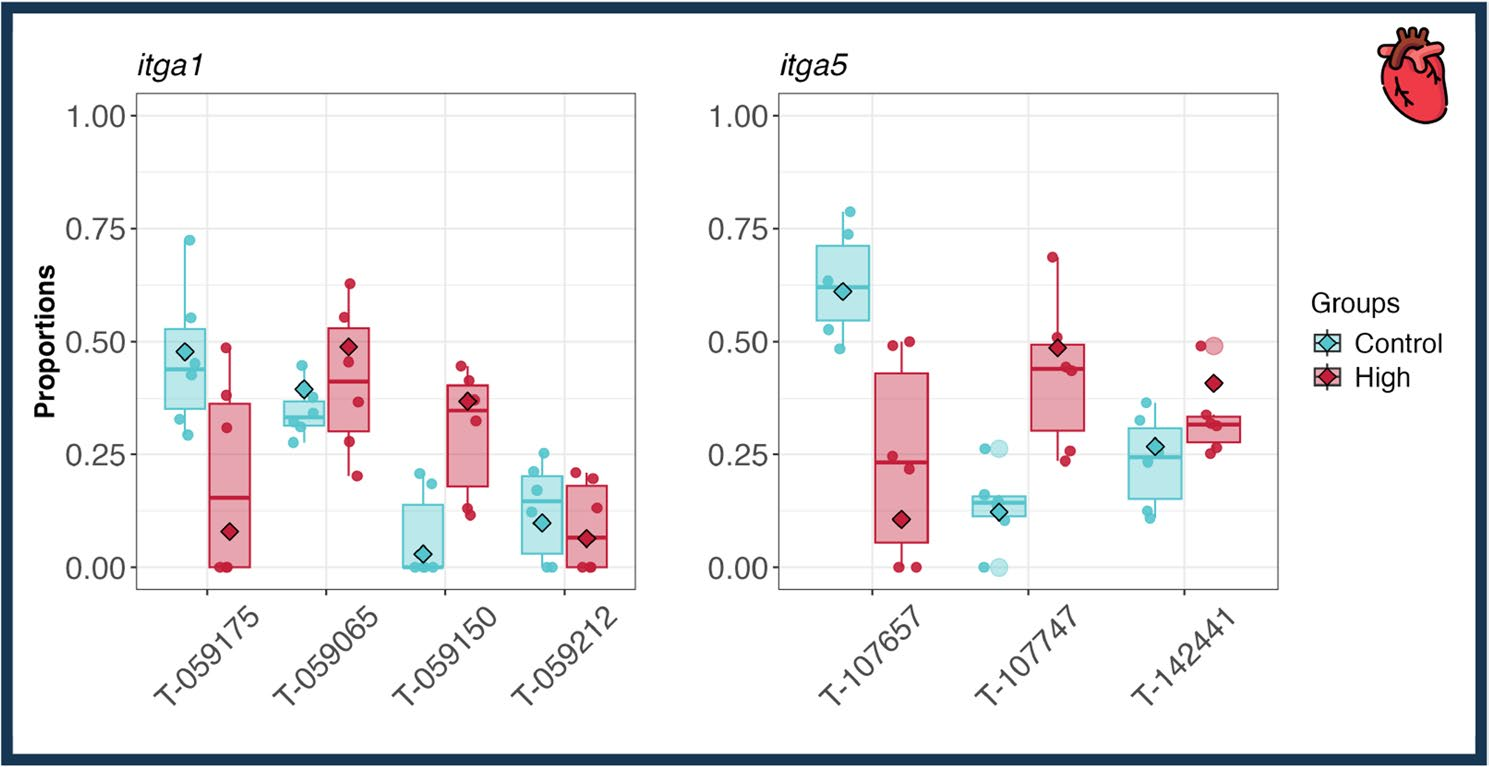
Two integrin alpha genes found to exhibit differential transcript usage in the heart. Blue boxes indicate the Control and red boxes indicate the High vitamin D salmon. DTU was observed in *itga1* and *itga5* in the heart. The transcript IDs are abbreviated with T- and the last six digits of the Ensembl ID. The proportion of *itga1* T- 059175 decreased from a mean of 0.48 to 0.08, while T- 059150 increased from a mean of 0.03 (Control) to 0.37 (High). The proportion of *itga5* T-107657 decreased from a mean of 0.61 (Control) to 0.11 (High), while T- 107747 increased from 0.12 (Control) to 0.49 (High)

As shifts in the relative usage of different isoforms within a gene can occur even without significant changes in overall gene or transcript level expression, we also performed a differential transcript usage analysis. In the filet, we identified 46 transcripts with altered isoform usage from 27 genes (Supplementary File 3.3). No genes that showed DTU in the filet were myofibrillar-associated genes. In the adductor mandibulae, we found 52 transcripts with altered usage patterns from 35 genes, none of which were myofibrillar-associated genes. In the stomach, 22 transcripts from 14 genes were found to exhibit differential transcript usage, none of which were myofibrillar or myofibrillar-associated genes. Again, the most profound impacts of vitamin D supplementation were found in the heart, where 398 transcripts of 258 genes showed significant changes in usage patterns. Four genes that showed DTU in the heart were myofibrillar-associated genes (Supplementary File 3.3), including *itga1* (integrin alpha 1) and *itga5* (integrin alpha 5) (Fig. 4).

### Differential alternative splicing events

**Fig. 5:**
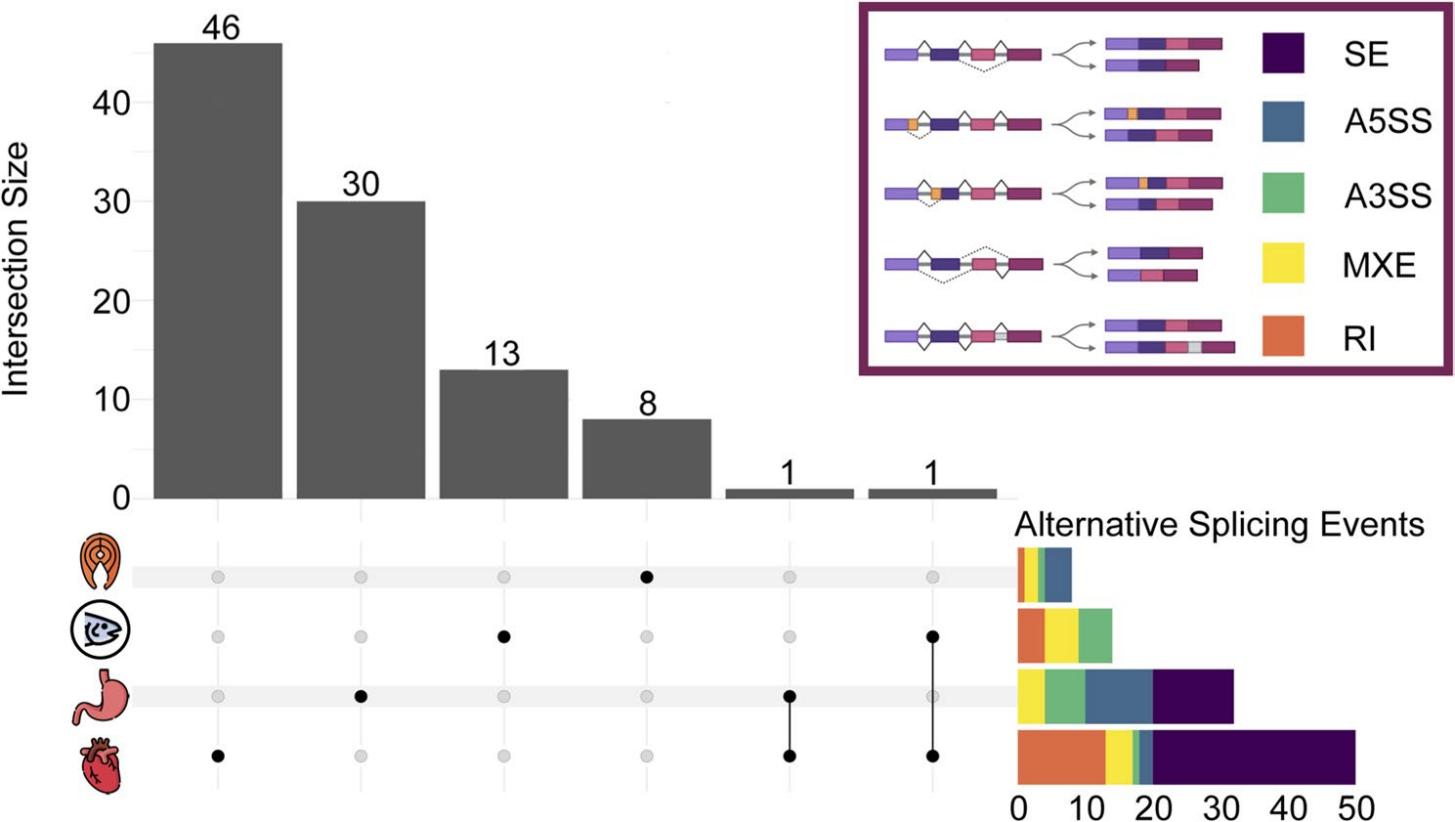
Upset plot detailing the number and type of alternative splicing events found to show Differential Alternative Splicing (DAS) in each muscle. The classes of splicing events detected are color-coded in the upper-right inset and the cumulative number of each type of event across all genes in a given muscle tissue are shown to the right of upset plot

We found that differential alternative splicing varied by muscle tissue (Supplementary File 3.4). Vitamin D had only minimal effects on alternative splicing in the filet (Fig. 5), where eight genes were alternatively spliced. In the adductor mandibulae, 14 genes were alternatively spliced (Fig. 5). Specifically, retained introns (RI) and alternative 3’ splice sites (A3SS) were increased in response to the high treatment. In the stomach, 31 genes were alternatively spliced and skipped exons (SE), alternative 3’ splice sites (A5SS), A3SS, and mutually exclusive exons (MXE) were all influenced by the vitamin D treatment (Fig. 5). Like the other levels of gene regulation, the largest impacts of vitamin D were seen in the heart where 48 genes were alternatively spliced (Fig. 5). Specifically, vitamin D seemed to increase SE and RI. Further, two genes were differentially alternatively spliced in more than one tissue, *tbc1d15* (tbc1 domain family member 15) in the stomach and heart, and *letm1* (leucine zipper-EF-hand containing transmembrane protein 1) in the adductor mandibulae and heart. The filet and adductor mandibulae each had one alternatively spliced myofibrillar-associated gene, *itga1* (integrin alpha 1) and *lmna* (lamin A/C), respectively. There were two alternatively spliced myofibrillar-associated genes in the stomach, *arvcf* (arvcf delta catenin family member) and *fhl5* (four and a half LIM domains 5). The heart had one alternatively spliced myofibrillar-associated gene, *fmnl3* (formin-like 3).

### Gene Ontology (GO) enrichment

**Fig. 6:**
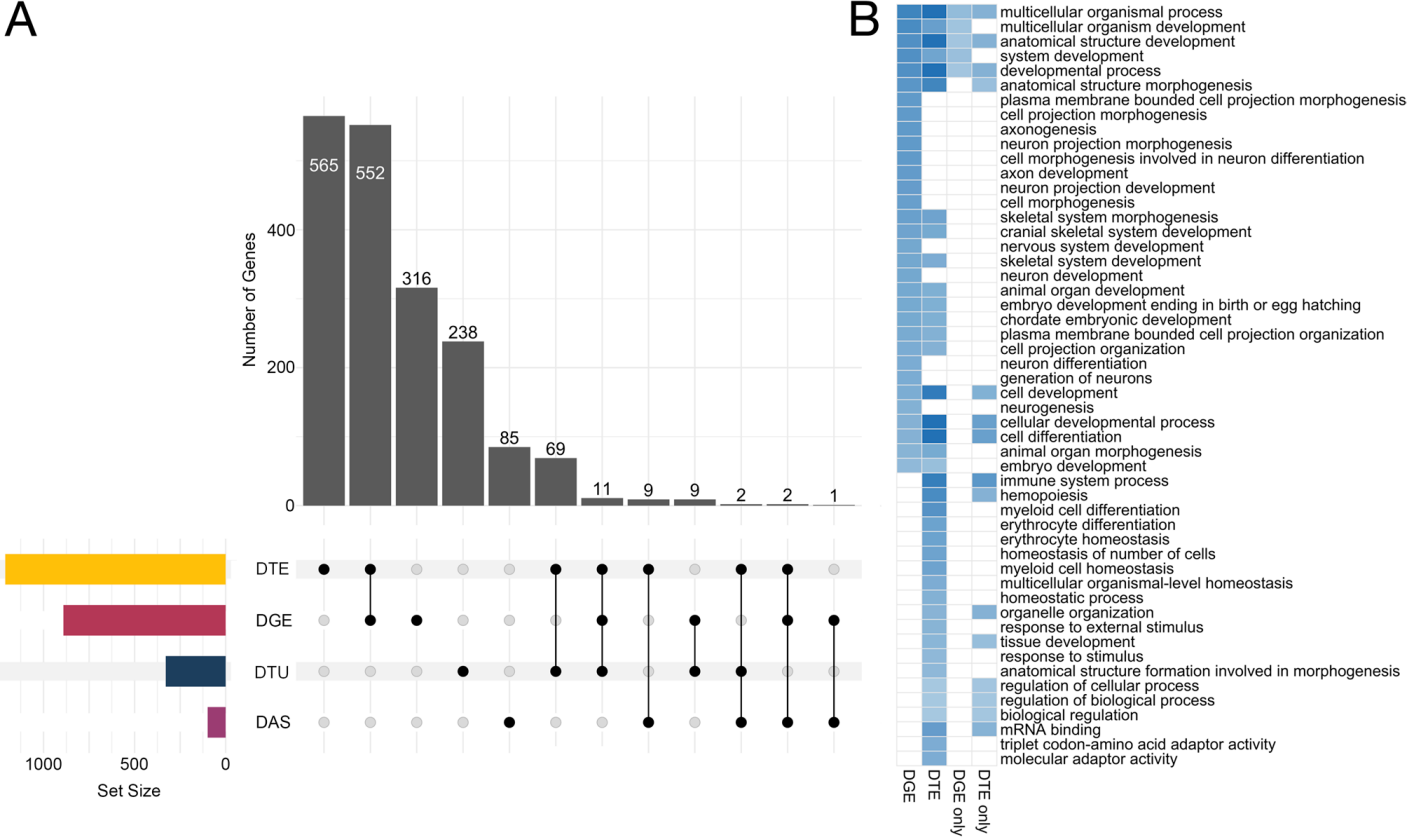
Summary of gene-level overlap and functional enrichment across transcriptomic analyses. **A** Upset plot showing the number of genes identified as significant in differential gene expression (DGE), differential transcript expression (DTE), differential transcript usage (DTU), and differential alternative splicing (DAS) analyses. Bars indicate the number of genes unique to each method or shared among methods. **B** Heatmap displaying enriched Gene Ontology (GO) terms for significant genes from DGE and DTE, as well as significant genes that were only present in DGE or DTE. Color intensity reflects the statistical significance of enrichment (-log_10_ p-value), with darker blue indicating stronger significance. White indicates that the GO category was not significantly enriched. The overlapping terms between DGE and DTE were dominated by general biological processes and metabolic regulation categories. DGE-only enrichments were limited to organismal processes and development, whereas DTE-only enrichments included immune system processes, tissue developmental pathways, and mRNA binding

We performed GO enrichment analyses on gene sets from the DGE and DTE analyses, as well as on genes unique to DGE or DTE. We identified 32 enriched GO terms for DGE, 39 for DTE, five for DGE only, and 15 for DTE only (Fig. 6). The DGE and DTE analyses shared a substantial portion of enriched categories, most of which were associated with general biological processes. The DGE only set contained only five enriched terms, associated with organismal processes and development. However, the DTE only set revealed a more diverse functional profile, including immune system process, tissue development, and mRNA binding. These terms are consistent with the ability of the transcript-level analysis to detect isoform-specific regulation, particularly in immune and developmental contexts. Collectively, these results demonstrate that while DGE and DTE capture a largely overlapping set of biological processes, each method detects unique functional signatures, with DTE showing the greater number and diversity of method-specific enrichments.

### Overlap between DGE, DTE, DTU, and DAS

There is substantial but far from total overlap between genes with DGE and DTE. In contrast, there was little overlap between DTU and either DGE or DTE (Fig. 6). Thus, analyses of differential expression are missing transcripts that are used differentially in response to vitamin D. There was also little overlap between genes that exhibit DAS and differential expression or transcript usage. Eleven genes were significant across DGE, DTE, and DTU analyses, including *aatm* (glutamic-oxaloacetic transaminase 2a), *mast4* (microtubule associated serine/threonine kinase family member 4), *pdha1a* (pyruvate dehydrogenase E1 component subunit alpha), *ptn* (pleiotrophin), and *slc25a12* (solute carrier family 25 member 12).

### Distribution of the number of isoforms in DGE, DTE, and DTU

**Fig. 7:**
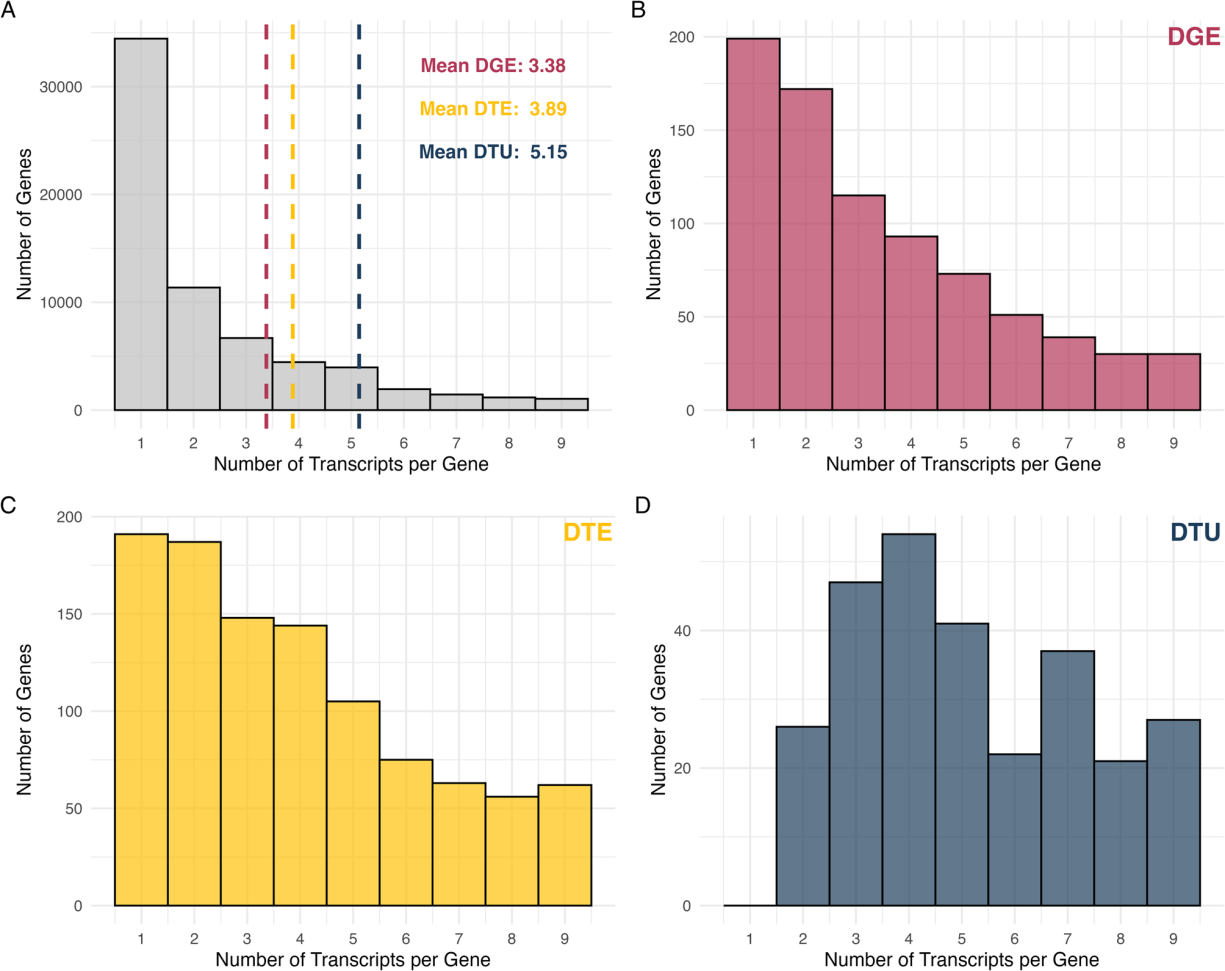
Distribution of the number of transcripts per gene for those detected to show different types of transcriptional changes across the four muscle types. **A** The distribution of the number of annotated isoforms in the Atlantic salmon Ensembl database across all genes expressed. **B** Distribution of the number of isoforms for genes with significant DGE. **C** Distribution of the number of isoforms for genes with significant DTE. **D** Distribution of the number of isoforms for genes with significant DTU. On average, genes with significant DGE (pink dotted line) tended to have fewer transcripts than genes with significant DTE (yellow dotted line) or DTU (blue dotted line)

The distribution of the number of isoforms per significant gene varied amongst the analyses (Fig. 7). The histogram of the number of isoforms per gene in the Atlantic salmon annotation file (Fig. 7A) revealed a right-skewed distribution, with most genes having one isoform. The number of isoforms per gene for the significant DGE genes showed a similar, but not as pronounced, right-skewed distribution (Fig. 7A, B). For DGE, the mean number of isoforms per gene was 3.38. Genes significant in the DTE analysis tended to have more isoforms than those significant in the DGE analysis (Fig. 7A, C). As genes must have more than one isoform to exhibit DTU, the number of isoforms per gene for the significant DTU genes was higher than for DGE or DTE (Fig. 7D), with a mean of 5.15 isoforms per gene.

### Vitamin D impacts on myofibrillar and myofibrillar-associated genes

**Fig. 8:**
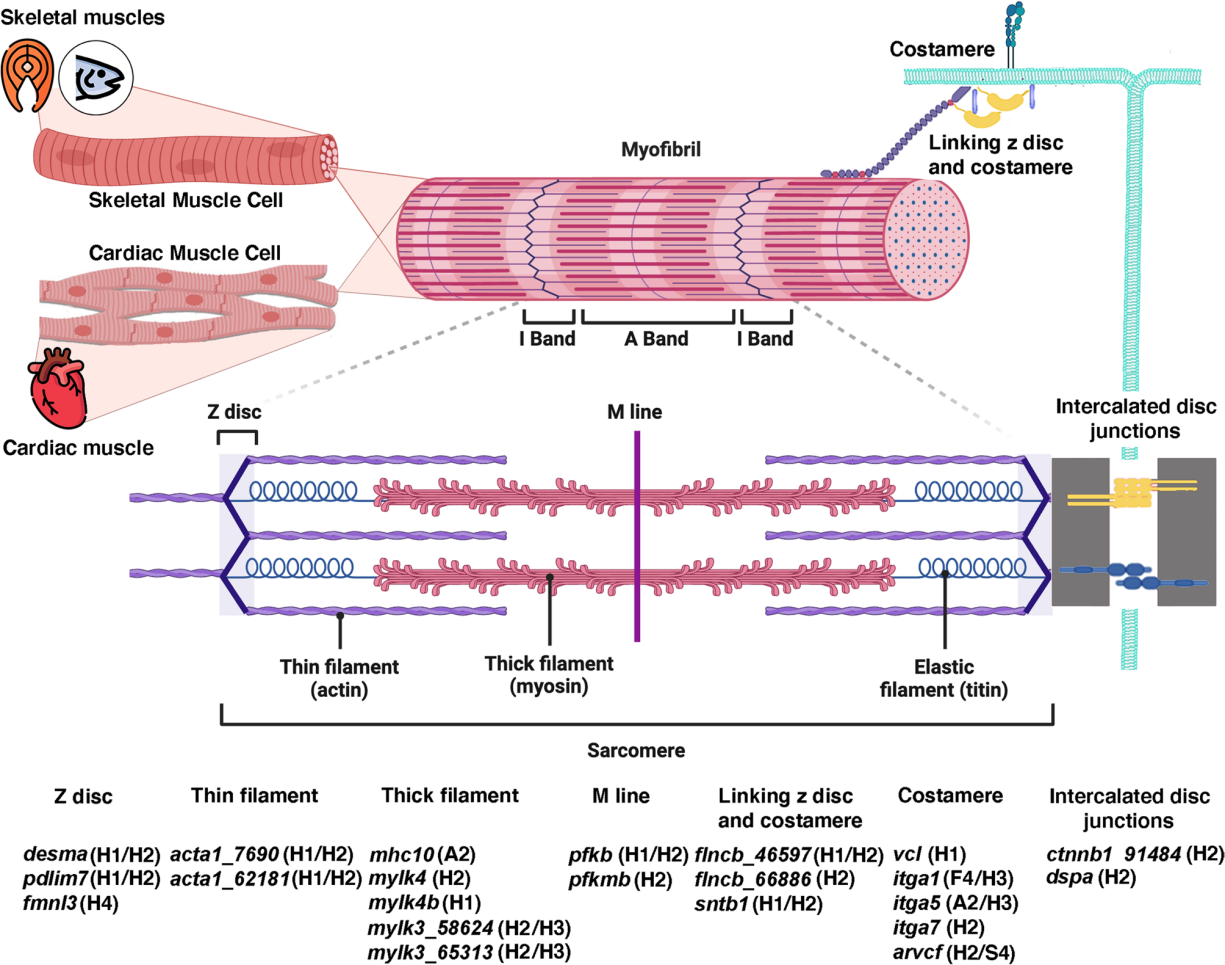
Myofibrillar and myofibrillar-associated proteins influenced by vitamin D supplementation. Both skeletal muscles, such as the filet and the adductor mandibulae, and cardiac muscle cells are striated due to the presence of muscle fibers containing tube-like myofibrils. These myofibrils are composed of sarcomeres arranged in series that are the basic muscle contractile units. Striated muscle sarcomeres are composed of thin (actin) filaments anchored to Z-discs and M-line attached thick (myosin) filaments that slide past actin filaments during sarcomere contraction. Myofibrils are attached to costameres that anchor them to the extracellular matrix. Intercalated discs, that are often orthogonal to the costameres in cardiomyocytes, contain many types of cell-to-cell junctions that link individual sarcomeres to more organ-wide cardiac contractions. The molecular proteins contributing to these structures are well-characterized in mammals [13]. Using mammals as a guide, we identified myofibrillar proteins that contribute to these particular muscle structures and are influenced by our vitamin D treatments. The tissues (H = Heart, F = Filet, A = Adductor mandibulae, and S = Stomach) and levels of differential transcription detected by different analytical methods (1 = DGE, 2 = DTE, 3 = DTU, and 4 = DAS) that were used to identify the changes in response to vitamin D are indicated. A large number of genes involved in forming the contractile machinery of striated muscle were directly affected in how they were expressed in response to vitamin D augmentation

Several known myofibrillar genes were impacted by vitamin D at every level of gene regulation investigated, including 12 that exhibited DGE, 20 found to have DTE, four that had significant DTU, and five with DAS. Eight myofibrillar genes were found in both DGE and DTE analyses: two copies of *acta1*, *desma*, *flncb*, *lmo2*, *pdlim7*, *pfkpa*, and *sntb1.* One myofibrillar gene, *arvcf*, was found in both DTE and DAS analyses. The myofibrillar genes showing transcriptional differentiation in response to vitamin D are located throughout the muscle cytoskeleton assembly (Fig. 8).

## Discussion

Vitamin D modulates salmon muscle transcription at multiple hierarchical levels. Transcriptional changes occur at the level of the gene, transcript, and splice junctions in all four muscle tissues. However, the impacts of vitamin D at all levels of gene regulation were particularly apparent in the heart. In the heart, we recovered transcriptomic changes that rivaled or far exceeded changes in all the other three muscles combined. Additionally, in contrast to the possibility that transcriptomic differentiation at these different levels were largely nested, we recovered little overlap among differential expression found at the gene, transcript, and splicing levels. Further, the number of isoforms per significant gene varied across DGE, DTE, and DTU analyses, indicating that each method tends to identify different sets of genes. Substantial impacts at the isoform level would likely be overlooked by solely analyzing expression differences at the gene level. Further, the identification of myofibrillar genes that are impacted by vitamin D at the gene, isoform, and splicing levels, demonstrates that vitamin D impacts on myofibrillar proteins could directly influence muscle contractions at multiple transcriptomic levels.

The exceptionally strong effect of vitamin D on transcription in the heart aligns with previous studies [10] and was also found at the isoform and splicing levels. The heart expresses a similar number of genes and transcripts as the other muscles (Table 2). However, the heart had around 10 times more tissue-specific transcripts than the other muscles (Table 2) which is consistent with the heart displaying more distinct and potentially specialized gene expression patterns compared to the other muscle tissues. This suggests that the outsized influence of vitamin D on the heart is not merely due to a greater number of differentially expressed genes but rather due to vitamin D having a significant regulatory role in multiple aspects of heart-specific transcriptome divergence.

Differential gene expression analysis alone does not robustly characterize the influence of vitamin D on the salmon transcriptome. Although there was surprisingly little overlap between the genes impacted by vitamin D at the gene, transcript, and splicing level, the greatest overlap among our analyses was observed between DGE and DTE. There were 565 genes inferred to be significantly differentially expressed at both the gene and DTE inferred transcript levels (Fig. 6). However, 645 genes exhibited significant DTE but non-significant DGE, including 12 myofibrillar or myofibrillar-associated proteins. The gene family of myosin light chain kinases in particular highlights some of the complexity at these different levels of expression. For example, two myosin light chain kinase 3 (*mylk3*) genes that are likely salmonid specific gene duplicates had differentially expressed transcripts but exhibited non-significant DGE in the heart (Fig. 3). Myosin light chain kinase 3 is thought to be cardiac-specific [59] and functions to phosphorylate myosin light chain 2 [60], which is an important structural protein that affects heart development and controls the rate and force of cardiac contraction [59, 61, 62]. Most of the effects of vitamin D on this critical heart gene were likely masked at the gene level due to divergent impacts of vitamin D on the multiple transcripts transcribed from each gene. In contrast, *mylk4b* was differentially expressed at the gene level but showed no differentiation at the transcript level. This is because while both *mylk4b* transcripts were downregulated in the high vitamin D treatment, their individual changes were not significant for DTE. Given the substantial impacts of vitamin D on several myosin light chain kinases, these genes could provide essential insight into how vitamin D plays a regulatory role in governing expression at multiple levels across vertebrate evolution.

Differential expression at the gene and transcript level had little overlap with genes that exhibited DTU, or shifts in how the relative proportions of different isoforms contribute to a single gene’s pooled mRNA. Of the 329 genes with significant DTU, 238 were only found in the DTU analyses, 69 were found in DTU and DTE, 11 were found in DTU, DTE, and DGE, nine were found in DTU and DGE, and two were found in DTU, DTE, and DAS (Fig. 6). Thus, examining shifts in isoform proportions provides insights into changes in expression that are largely distinct from expression inferred for the whole gene region. Of the genes that were found to use isoforms differently, four represented myofibrillar-associated genes, including two copies of *mylk3* as well as *itga1* and *itga5* in the heart (Fig. 4). None of these were identified in the DGE analysis. Vitamin D influencing these isoform proportions could have substantial consequences for cardiac muscle function. For instance, integrin alpha subunits, such as *itga1* and *itga5*, are expressed in vertebrate cardiomyocytes and function in cardiac morphogenesis, endocardial differentiation, and remodeling after injury [63–66]. Vitamin D is acting on the isoforms of these critical muscle genes in ways that would not be revealed through simply examining DGE alone.

Contrary to our expectations, genes that were identified to exhibit single alternatively spliced junctions in response to vitamin D had little overlap with genes that showed DGE, DTE, or DTU. Of the 99 genes inferred to exhibit alternative splicing junctions, 85 were identified in only the DAS analyses, nine were also identified in DTE, two in DTE and DGE, and one in DGE (Fig. 6). Differential alternative splicing also varied amongst the muscle tissues. Skeletal muscle had the fewest alternative splicing events, with eight in the filet and 14 in the adductor mandibulae. Further, neither skeletal muscle had any exon skipping (SE) events, even though SE is the most common alternative splicing event in vertebrates [67, 68]. The stomach had 31 differential splicing events split amongst all event types except RI, the least frequent alternative splicing event in vertebrates [67]. The heart showed the greatest effect of vitamin D on alternative splicing, with 48 events, most of which were SE and RI. The diversity of responses to vitamin D shown here could have implications for tissue-specific adaptations at the isoform level. It should be investigated further whether there could also be different patterns of gene-splicing in response to vitamin D than what is generally observed and that dominate in different types of muscles.

Vitamin D impacts the expression of many myofibrillar proteins in salmon that influence muscle contractions, and this has general implications for vitamin D’s role in vertebrate heart function. It is well-known that vitamin D is essential for musculoskeletal health [69, 70] and there are clear links between this nutrient and cardiovascular functioning [71]. However, it is generally unclear whether the whole-organism effects of vitamin D are often more indirect via systemic changes such as in circulating calcium and phosphate levels [70], via impacts on mitochondrial function that might be particularly critical in the heart [17, 19], or more generally on the structural elements involved in heart contraction. Importantly, we found that vitamin D directly modulates a number of structural myofibrillar and myofibrillar-associated genes at multiple hierarchical levels of gene regulation (i.e., gene, transcript, and splicing events), suggesting that vitamin D has a direct regulatory role in muscle contractile physiology. As myofibrillar proteins are essential to muscle development, growth, and repair, how we move, and whether our heart continues beating, these findings have important implications for vitamin D’s general role in optimizing how muscles could influence whole-organism well-being.

Our examination of differential expression at multiple levels of gene regulation has provided critical insights into how vitamin D simultaneously impacts multiple types of muscle. We have also provided a practical examination of multiple methods of analyzing bulk RNA-seq data and demonstrated cases where less commonly recommended analyses (e.g., DTE) could be useful. As myofibrillar proteins are known for having multiple isoforms with divergent effects [13, 15, 16], a focus on these proteins provides a good opportunity for the evaluation of these tools. Generally, the literature recommends the combination of DGE and DTU for bulk RNA-seq data, as these two approaches when implemented might encapsulate transcriptional diversity [44]. However, we found much less overlap between DTE and DGE than we would have expected (Fig. 6). Some of this is likely due to the idiosyncrasies of the methods [72]. However, this is also likely due in large part to several genes impacted by vitamin D having transcripts that are differentially expressed in opposing directions, like the myosin light chain kinases (Fig. 3). Further, while DTU generally implies DTE [72], we found that this was not always the case and the majority of genes detected by a DTU analysis in our data were not detected in the DTE analysis (Fig. 6). This discrepancy in detection likely occurred when the expression differences of uncommon isoforms are not large enough to be detected by DTE but are detected by the more sensitive DTU analysis (see Fig. 1), an issue that is likely exacerbated as the number of isoforms per gene increases (Fig. 7).

Alternative splicing and isoform variation among different tissue types and under different natural conditions such as during nutrient enrichment is undoubtedly biologically important. Although the degree to which alternative transcripts yield functionally relevant protein isoforms remains under investigation [73], it is clear that splicing can contribute to functional protein diversity and regulate protein expression levels [74–76]. These processes appear to be particularly relevant in muscle [11], where alternative isoforms may modulate gene signalling, localize to specific cell types, and influence protein stability [42]. Consequently, many transcriptomic shifts detected as DTE, DTU, and DAS could have functional effects, especially across muscle tissues. Dissecting the responses of uncommon isoforms to stimuli like vitamin D may be particularly critical to understanding the role of essential nutrients on health as small changes in lowly expressed isoforms might often have an outsized role in disease pathologies. For instance, although skeletal muscle actin (*ACTA1*) represents only 20% of the total actin pool in human cardiomyocytes, small amounts of a unique *ACTA1* isoform in the heart can cause cardiomyopathy via disruption of molecular contractility through structural alterations of this gene’s interactions with tropomyosin [77]. When trying to identify how genes with multiple divergent isoforms, like myofibrillar proteins, respond to a stimulus like vitamin D across a range of tissue types, it is worthwhile to consider the inferences that can now be made at multiple hierarchical levels of transcriptomic divergence.

## Conclusions

Vitamin D modulates salmon muscle transcription at the level of the gene, transcript, and splice junctions in all four muscle tissues, with particularly strong effects seen in the heart. The tissue-specific responses we observed at all levels of transcriptomic differentiation indicate that findings from vitamin D’s effects on well-studied skeletal muscle are not likely to be directly generalizable to other muscle types. Further, the minimal overlap among significant genes found at the gene, transcript, and splicing levels suggests examining only DGE can mask important genes that show differential expression at other levels. For example, we found that several myosin light chain kinase isoforms were particularly impacted by vitamin D in the heart, but many of the genes exhibited isoforms that were differentially expressed in opposing directions. This example is striking as it suggests a conserved regulatory role of vitamin D in muscle across vertebrate evolution that includes multiple gene duplication events that would not become apparent in only a whole gene level analysis. Our identification of myofibrillar genes that are impacted by vitamin D at all levels of gene regulation further demonstrate that vitamin D likely directly impacts the proteins that influence muscle contractions. Together, these findings help clarify how vitamin D influences muscle health, function, and transcription across multiple levels.

## Supplementary Information

Supplementary Material 1: Images of a representative dissection.

Supplementary Material 2: myofibrillar_genes.xlsx. Spreadsheet containing a list of myofibrillar and myofibrillar-associated genes in Atlantic salmon.

Supplementary Material 3: Tabs 1-4 include lists of significant genes and transcripts from the DGE, DTE, DTU, and DAS analyses.

## Data Availability

The RNA-seq data are available at the NCBI Sequence Read Archive under accession number PRJNA1160017.

## Abbreviations

DGE: Differential Gene Expression
DTE: Differential Transcript Expression
DTU: Differential Transcript Usage
DAS: Differential Alternative Splicing
SE: Exon Skipping
A5SS: Alternative 5’ Splice Site
A3SS: Alternative 3’ Splice Site
MXE: Mutually Exclusive Exons
RI: Intron Retention
