# Supplementary material for: Vitamin D elicits tissue-specific isoform expression in Atlantic salmon muscles
Source: BMC Genomics. 2025 Nov 12;26:1031. doi: 10.1186/s12864-025-12209-1 (PMC12613645; doi:10.1186/s12864-025-12209-1)
Supplement: Supplementary file 1 — Supplementary Material 1: Images of a representative dissection. [file 12864_2025_12209_MOESM1_ESM.pdf]

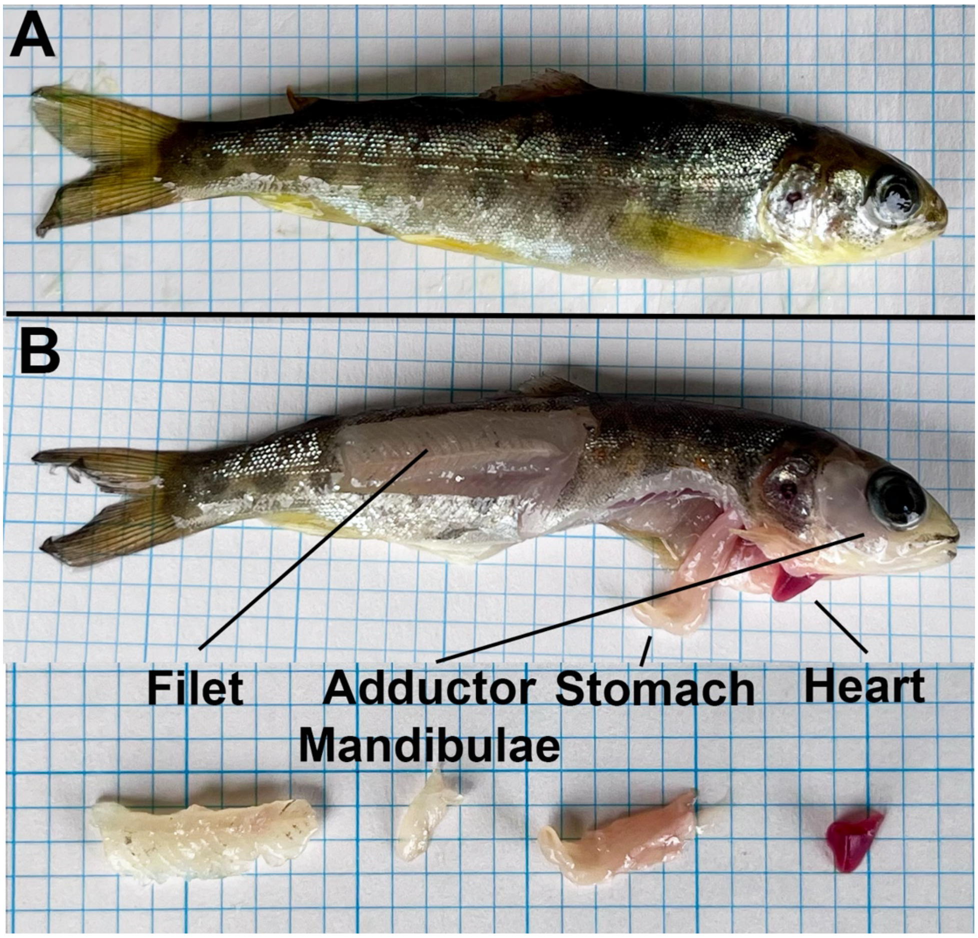

FigS. 1. Images of a representative dissection. An example salmon from the study prior to dissection. The same salmon with representative clean dissections of structures representing the four muscle types investigated: 1. salmon filets (axial skeletal muscle), 2. the adductor mandibulae (craniofacial skeletal muscle), 3. the stomach (smooth muscle), and 4. heart (cardiac muscle).
